# The effect of non-small cell lung cancer histology on survival as measured by the graded prognostic assessment in patients with brain metastases treated by hypofractionated stereotactic radiotherapy

**DOI:** 10.1186/s13014-016-0667-x

**Published:** 2016-07-13

**Authors:** Liang-Hua Ma, Guang Li, Hong-Wei Zhang, Zhi-Yu Wang, Jun Dang, Shuo Zhang, Lei Yao

**Affiliations:** Department of Radiation Oncology, The First Affiliated Hospital of China Medical University, 155 North Nanjing Street, Heping District, Shenyang, 110001 Liaoning China

**Keywords:** Brain metastases, Non-small cell lung cancer, Graded prognostic assessment, Histology, Hypofractionated stereotactic radiotherapy

## Abstract

**Background:**

The purpose of this study was to investigate the impact of histology on survival stratified by the Graded Prognostic Assessment (GPA) for non-small cell lung cancer (NSCLC) in a group of selected patients treated recently.

**Methods:**

A total of 171 NSCLC patients with brain metastases treated by hypofractionated stereotactic radiotherapy with or without whole-brain radiotherapy between 2001 and 2011 were included. The GPA score was calculated for each patient. Tumor histologies were categorized into adenocarcinoma (ADCA) and non-ADCA. Median survival time (MST, in months) was calculated using the Kaplan-Meier method. The log-rank test was used to determine statistical differences.

**Results:**

MSTs by histology were: ADCA 15 (*n* = 92) and non-ADCA 10 (*n* = 79) (*p* < 0.001). For all patients, the MSTs by GPA score were: GPA 3.5-4, 24; GPA 2.5-3, 15; GPA 1.5-2, 9 and GPA 0-1, 6 (*p* < 0.001). The histology of ADCA showed a statistically significant higher MST than non-ADCA for patients with GPA 2.5-4. For GPA 2.5-3, MSTs were: ADCA 18, non-ADCA 10 (*p* = 0.007); for GPA 3.5-4, MSTs were: ADCA 30, non-ADCA 17 (*p* = 0.046). For GPA 0-2, MSTs did not differ significantly by histology. For GPA 0-1, MSTs were: ADCA 8, non-ADCA 4 (*p* = 0.146); GPA 1.5-2, MSTs were: ADCA 10, non-ADCA 8 (*p* = 0.291). We further found that non-ADCA in upper GPA class (3.5–4) had similar survival with ADCA in lower GPA class (2.5–3) (MSTs were 17 and 18, respectively, *p* = 0.775). This phenomenon also happened between patients of non-ADCA in upper GPA class (2.5–3) and those of ADCA in lower GPA class (1.5–2) (MSTs were both 10, *p* = 0.724).

**Conclusions:**

We confirmed that the histology of NSCLC had effect on the GPA in these selected patients treated recently. ADCA showed a statistically significant higher MST than non-ADCA with GPA 2.5-4. The non-ADCA in upper GPA classes (3.5-4 and 2.5-3) had similar survival to ADCA in lower GPA classes (2.5-3 and 1.5-2, respectively). The histology as a new factor should be added to the original GPA for NSCLC.

## Background

Today, it is clear that the prognostic factors are different in patients with brain metastases. To predict those who have better survival in all patients, many different prognostic indices have been developed [1–5]. The Graded Prognostic Assessment (GPA) is a newer prognostic index [4] and is less subjective and more quantitative than the widely used Recursive Partitioning Analysis (RPA) [1]. It incorporated number of brain metastases which was of proven significance in the RTOG 9508 trail and excluded the estimation of primary tumor control which is subjective and difficult to quantify. The GPA was refined with diagnosed-specific prognostic indices for patients with brain metastases from some different site or histology [6, 7]. The non-small cell lung cancer (NSCLC) specific GPA index was a 4-titered prognostic index. It is the sum of scores (0, 0.5, and 1.0) for four factors (age, Karnofsky Performance Scale (KPS), number of brain metastases and presence of extracranial metastases) (Table 1) [6, 7]. The histology of NSCLC as an independent prognostic factor for patients with brain metastases had been reported [8–10], while its effect on the GPA index was not well defined. Guo et al. analyzed a group of unselected patients with NSCLC brain metastases treated from 1982 to 2004, they found that adenocarcinoma (ADCA) had a better survival time than other histologies for patients with GPA 0-3 [11]. Although the study of Guo et al. makes us notice the effect of histology on the GPA, there are still some questions need to be answered. 1. The conclusions of Guo et al. are based on the patients treated in an earlier treatment period (1982–2004), whether these conclusions changed in recent years? 2. Whether these conclusions still exist in selected patients? 3. Whether the histology has other effects on the GPA? To answer these questions and further investigate the impact of histology on survival stratified by the GPA we analyzed a group of selected patients with brain metastases from NSCLC treated recently in our institution.

**Table 1:** Details of the GPA for NSCLC [6, 7]

	GPA score		
	0	0.5	1.0
Age	>60	50–60	<50
KPS	<70	70–80	90–100
No. of brain metasases	>3	2–3	1
Extracranial metastases	present	-	absent

## Methods

### Patient population

The present study was based on the data from patients with brain metastases treated by hypofractionated stereotactic radiotherapy (HSRT) with or without whole-brain radiotherapy (WBRT) in our institution. Those newly diagnosed patients from pathologically confirmed NSCLC were included. These patients did not receive other treatments before their brain metastases were diagnosed. From April 2001 to September 2011, 171 patients were included in this study. There were 92 patients with ADCA and 79 patients with non-ADCA (squamous cell carcinoma (SCC): 49, large-cell carcinoma (LCC): 10, other styles: 20). The characteristics are listed in Table 2.

**Table 2:** Patient and treatment characteristics

	Number of patients (*n* = 171)	%
Histology		
ADCA	92	53.8
Non-ADCA	79	46.2
Gender		
Male	98	57.3
Female	73	42.7
GPA class		
0–1	16	9.4
1.5–2	63	36.8
2.5–3	69	40.4
3.5–4	23	13.4
Age		
< 50	38	22.2
50–60	50	29.2
> 60	83	48.6
KPS		
< 70	32	18.7
70–80	82	48.0
90–100	57	33.3
No. of brain metastases		
1	83	48.6
2–3	57	33.3
> 3	31	18.1
Extracranial metastase		
Present	45	26.3
Absent	126	73.7
RPA		
I	47	27.5
II	92	53.8
III	32	18.7
Treatment		
HSRT	54	31.6
HSRT + WBRT	117	68.4

### HSRT

The HSRT process has been described before [12] and as follows. A non-invasive mask was used to immobilize patients. The helical computed tomography and magnetic resonance images were fused by image fusion software. The gross tumor volume (GTV) was defined as the contrast-enhanced tumor. The planning tumor volume (PTV) was defined by adding a 1-mm margin to the GTV. Brainlab’s stereotactic treatment planning system and SCAN 4.05 version of the three-dimensional positioning and target system were applied to calculate dose. The prescribed dose was delivered to the 80–90 % dose line, which included more than 98 % of the PTV. The common dose was 32Gy/4f, delivered every other day. This method of dose administration was based on two previous studies [13, 14]. Of course, the dose prescription and fractions varied due to the size, location, number of lesions and whether WBRT was given. Irradiation was performed with 6-MV photons from a liner accelerator (Siemens PRIMUS-M Germany) using multiple non-coplanar arcs. HSRT was performed alone in 54 patients and was combined with WBRT in 117 patients. For those patients treated combining with WBRT (planning dose was 40Gy/20f, 5f/week) the time interval was within 1 week before or after HSRT.

### Statistical analysis

The GPA score was calculated for each patient. Tumor histologies were categorized into ADCA and non-ADCA. Overall survival was estimated using the Kaplan-Meier method. Median survival time (MST, in months) was calculated from the date of brain metastases diagnosis to the date of death. The log-rank test was used to determine whether significant survival differences were present among patient groups. A *p* value <0.05 was considered statistically significant. Analyses were performed using SPSS, version 13.0 (SPSS Inc).

## Results

At the end of December 2014, 155 patients (90.6 %) had died. For 171 patients, the median follow-up time was 13 months (range 1–116 months). The MST for all patients was 13 months (95 % CI 11.258–14.742 months). The median age was 59 years (range 25–80 years). The median KPS was 80 (range 60–100). The median number of lesions at diagnosis was 2 (range 1–8).

**Fig. 1:**
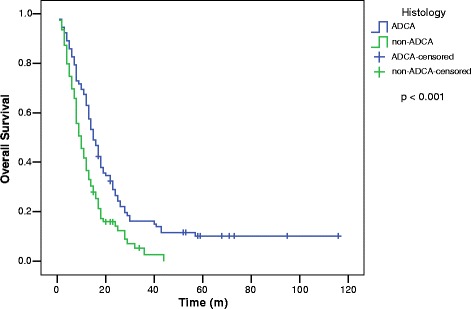
Overall survival for patients with brain metastases from non-small cell lung cancer by histology

**Fig. 2:**
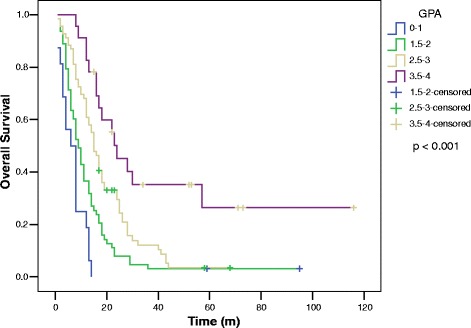
Overall survival for patients with brain metastases from non-small cell lung cancer by graded prognostic assessment group

MSTs by histology were: ADCA 15 (*n* = 92) and non-ADCA 10 (*n* = 79) (*p* < 0.001) (Fig. 1). For all patients, the MSTs by GPA score were: GPA 3.5-4, 24; GPA 2.5-3, 15; GPA 1.5-2, 9 and GPA 0-1, 6 (*p* < 0.001) (Fig. 2).

**Fig. 3:**
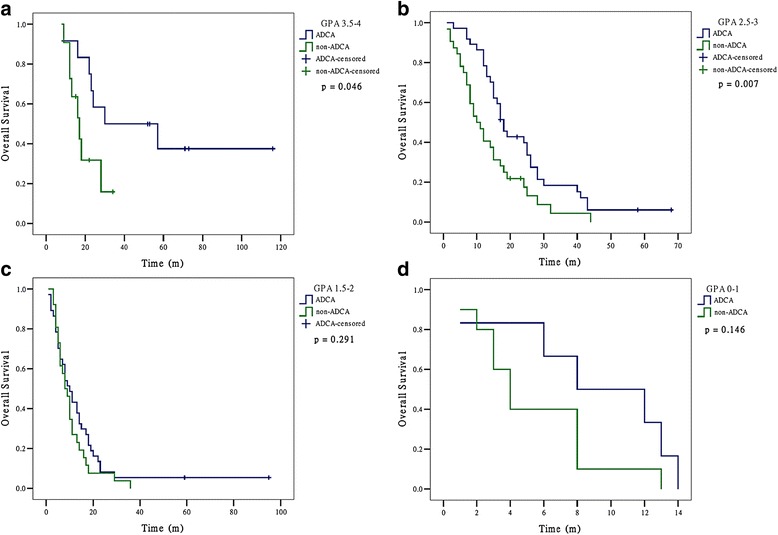
Overall survival for patients with brain metastases from non-small cell lung cancer by histology and by graded prognostic assessment group

The histology of ADCA showed a statistically significant higher MST than non-ADCA for patients with GPA 2.5-4. For GPA 2.5-3, MSTs were: ADCA 18, non-ADCA 10 (*p* = 0.007); for GPA 3.5-4, MSTs were: ADCA 30, non-ADCA 17 (*p* = 0.046). For GPA 0-2, MSTs did not differ significantly by histology. For GPA 0-1, MSTs were: ADCA 8, non-ADCA 4 (*p* = 0.146); GPA 1.5-2, MSTs were: ADCA 10, non-ADCA 8 (*p* = 0.291) (Fig. 3).

**Fig. 4:**
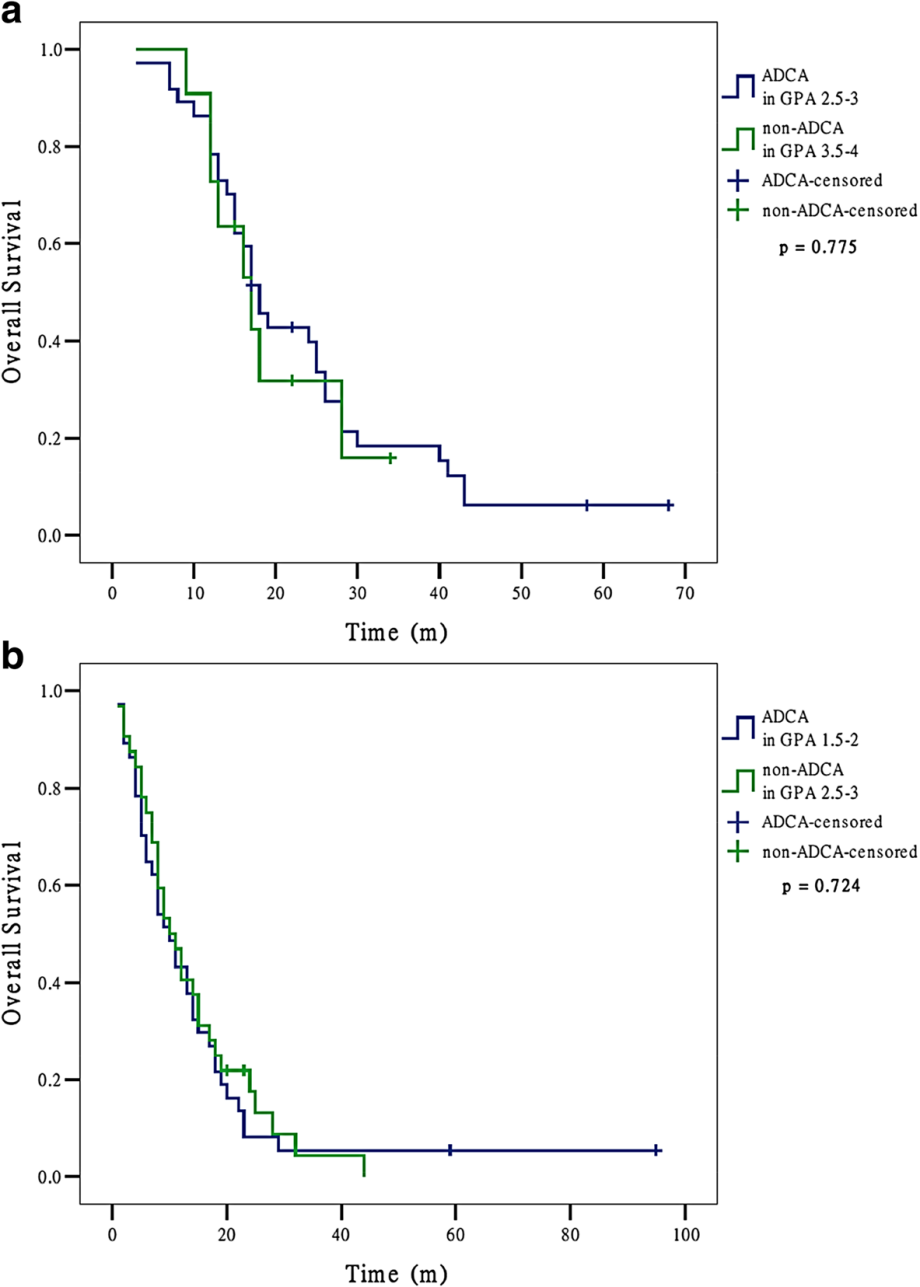
Overall survival for patients with brain metastases from non-small cell lung cancer who had similar median survival time

We further found that non-ADCA in upper GPA class (3.5-4) had similar survival with ADCA in lower GPA class (2.5-3) (MSTs were 17 and 18, respectively, *p* = 0.775). This phenomenon also happened between patients of non-ADCA in upper GPA class (2.5-3) and those of ADCA in lower GPA class (1.5-2) (MSTs were both 10, *p* = 0.724) (Fig. 4).

## Discussion

Although the GPA has been validated in different NSCLC groups [6, 7, 15, 16], the effect of histology on this index was not realized until recently.

Nieder et al. analyzed 209 patients with brain metastases from NSCLC treated with different methods (radiosurgery, surgical resection, WBRT, best supportive care) and found that ADCA histology is an independent prognostic factor in different multivariate models [8]. They also analyzed the histology and the GPA index together in a multivariate model and found that both factors remain statistically significant [8]. In their study the patients with ADCA had significantly longer MST than those with non-ADCA (SCC or LCC). In a previous study which included the same patient population of the present study we found that histology (non-ADCA vs ADCA) was an independent prognostic factor (HR 1.606 95 % CI 1.138–2.267, *p* = 0.007) besides those factors in the GPA (age, KPS, number of brain metastases and presence of extracranial metastases) [12]. These results suggested that histology of NSCLC should be taken into account when using the GPA index.

Guo et al. evaluated the impact of histology on survival of patients with NSCLC brain metastases stratified by the GPA. They analyzed a group of unselected patients (treated by WBRT, radiosurgery, or surgery) in a earlier period (1982–2004) and found that ADCA had a statistically significant higher MST than other histologies in patients with GPA 0-3 [11]. In the present study we observed a similar phenomenon in our selected patients (treated by HSRT with or without WBRT) in recent years (2001–2011), but the survival advantage of ADCA only existed in patients with GPA 2.5-4. Although there were some differences between the present study and that of Guo et al., both studies had a similar conclusion: the patients of ADCA had a statistically significant longer survival time than other histologies of NSCLC in some GPA classes. We further found that non-ADCA in two upper GPA classes (3.5-4 and 2.5-3) had similar survival to ADCA in their lower GPA classes (2.5-3 and 1.5-2, respectively). Our results suggested that the histology as a new factor should be added to the original GPA for NSCLC. For example, if ADCA is set to 1 and non-ADCA is set to 0, the patients of non-ADCA with original GPA 3.5-4 and those patients of ADCA with original GPA 2.5-3 will have the same new score 3.5-4, at the same time the patients of ADCA with original GPA 3.5-4 will have the new score 4.5-5, then not only these patients who had better survival could be distinguished but also those who had similar MST will be merged. Because our suggestion was based on a single-institution retrospective study, whether it is possible to use the adapted GPA score in other patient populations needs to be further investigated.

Many studies had reported that ADCA had better survival than other subtypes of NSCLC [8–10, 17], while the reasons are still controversial. One main controversy is whether new systemic drugs, especially targeted therapies, are causes for the survival advantage of ADCA. In a study which included unselected NSCLC brain metastases patients treated with different methods between 1990 and 2011, Nieder et al. found that patients with ADCA had significantly longer survival (*p* = 0.019, the absolute difference in MST was 0.5 months) even in an earlier period (1990–2003) when pemetrexed and EGFR tyrosine kinase inhibitors were not available [8]. The absolute differences became much larger later (2004–2011). MST in patients with ADCA increased from 2.5 to 6.8 months (*p* = 0.039). In SCC, an increase from 2.0 to 3.3 months could be observed (*p* = 0.430) [8]. Kuremsky et al. analyzed the data of patients treated with Gamma Knife radiosurgery between 2000 and 2010 and found that ADCA had a survival advantage over SCC (MSTs were 10.2 and 5.3 months, respectively, *p* = 0.008) [9]. To further investigate the possible causes for this difference, they compared the outcomes for patients treated before and after 2005. Their results showed no differences in the rates of local control (*p* = 0.58), distant brain failure (*p* = 0.48), or the overall survival (*p* = 0.64) between the two cohorts [9]. We also analyzed the possible effect of treatment periods on the survival advantage of ADCA and got similar results to that of Kuremsky et al. In non-ADCA there was no difference of survival time between two treatment periods (10 months for 2001–2004 and 9 months for 2005–2011, *p* = 0.593). Although an increase of MST from 13 to 17 months was observed in ADCA since 2005, there was no statistically significance (*p* = 0.090).

Finally, the limitations of our study should be mentioned: 1. As a single-institution study the sample size is small; 2. The retrospective nature of this study is prone to bias; 3. We could not confirm the causes of survival advantage of ADCA for absence of data on gene mutations. We suggest that multi-institution prospective studies be performed to further assess the effect of histology and to confirm the causes for this effect in the future.

## Conclusions

We confirmed that the histology had effect on the GPA in these patients with brain metastases from NSCLC treated by HSRT with or without WBRT. ADCA showed a statistically significant higher MST than non-ADCA with GPA 2.5-4. The non-ADCA in upper GPA classes (3.5-4 and 2.5-3) had similar survival to ADCA in lower GPA classes (2.5-3 and 1.5-2, respectively). The histology as a new factor should be added to the original GPA for NSCLC.

## Abbreviations

ADCA, adenocarcinoma; GPA, Graded Prognostic Assessment; GTV, gross tumor volume; HSRT, hypofractionated stereotactic radiotherapy; KPS, Karnofsky Performance Scale; LCC, large-cell carcinoma; MST, Median survival time; NSCLC, non-small cell lung cancer; PTV, planning tumor volume; RPA, Recursive Partitioning Analysis; SCC, squamous cell carcinoma; WBRT, whole-brain radiotherapy
